# The Impact of 8 Weeks of Combined Physical Exercise Training on SIRT3 and mTOR in Lymphocytes, and on Lipid Peroxidation

**DOI:** 10.3390/healthcare12030350

**Published:** 2024-01-30

**Authors:** Jorge Pinto Soares, Ricardo Cardoso, Vanessa Almeida, Ana Fátima Pereira, Amélia M. Silva, Maria Paula Mota

**Affiliations:** 1 Research Centre in Sports Sciences, Health, and Human Development (CIDESD), 5001-801 Vila Real, Portugal; mpmota@utad.pt; 2 Department of Sport of Science Exercise and Health, School of Life and Environmental Sciences (ECVA), University of Trás-os-Montes and Alto Douro (UTAD), 5000-801 Vila Real, Portugal; 3 Centre for the Research and Technology of Agro-Environmental and Biological Sciences (CITAB), University of Trás-os-Montes and Alto Douro (UTAD), 5000-801 Vila Real, Portugal; ricardo19moc@gmail.com (R.C.); vvanessaalmeida@gmail.com (V.A.);; 4 Polytechnic Institute of Setubal (IPS), 2910-761 Setúbal, Portugal; ana.fatima.pereira@ese.ips.pt; 5 Department of Biology and Environment, University of Trás-os-Montes and Alto Douro (UTAD), 5000-801 Vila Real, Portugal

**Keywords:** physical exercise, SIRT, mTOR, oxidative stress, aging, lymphocytes

## Abstract

The sirtuins (SIRT) protein family and the mechanistic/mammalian target of rapamycin (mTOR) are intracellular molecules that have been involved in the regulation of several biological processes, as well as in various aging-related processes. This pilot study, in small scale, aimed to analyze the effects of an 8-week physical exercise program on SIRT3 and mTOR levels in lymphocytes, as well as on lipid peroxidation in middle aged and older men. A total of 9 participants aged between 56 and 73 years were enrolled in an 8-week physical exercise program comprising cardiovascular and high-intensity interval training. The program involved three sessions per week, each lasting 45–60 min, conducted on non-consecutive days. Tests were conducted before and after the experimental period (pre- and post-training). Assessments included a vertical jump, 20 m velocity, ball throwing, and an aerobic capacity test. Lipid peroxidation (MDA) was measured in plasma as an oxidative stress biomarker. Additionally, sirtuin 3 (SIRT3/β-actin) and mTOR (mTOR/β-actin) levels were measured in isolated lymphocytes extracted from venous blood. Following the exercise training period, our results demonstrated a significant improvement in aerobic capacity (pre-training: 615.4 ± 45.3 m; post-training: 687.2 ± 34.6 m; t = −2.521; *p* = 0.012) and 20 m velocity (pre-training: 4.6 ± 0.5 s; post-training: 4.3 ± 0.3 s; t = −2.023; *p* = 0.04). Concerning blood variables, there was a significant decrease in mTOR levels (pre-training: 0.857 ± 0.593; post-training: 0.214 ± 0.097; t = −2.547; *p* = 0.011), while no changes were observed in SIRT3 (pre-training: 0.608 ± 0.404; post-training: 0.516 ± 0.390; t = 0.533; *p* = 0.594) and MDA (pre-training: 8420 ± 4615; post-training: 8800 ± 3163; t = −0.533; *p* = 0.594). The notable reduction in mTOR levels in lymphocytes following the 8-week physical exercise program suggests a potential role of exercise in modulating immune cell dynamics, particularly in middle-aged and older individuals. Furthermore, the exercise regimen resulted in improvements in physical function, including enhanced aerobic capacity and walking velocity.

## 1. Introduction

In the management of metabolic stress and energy balance in mammals, sirtuins (SIRT) and the mechanistic/mammalian target of rapamycin (mTOR) are recognized to play an essential role. Certainly, these two intracellular molecules have been implicated in various aging-related processes, including cellular senescence, autophagy, immune responses, cell stem regulation, protein metabolism, and mitochondrial function [1,2,3,4,5]. Considering all of this, several works have emerged in order to understand the underlying mechanisms and possible factors that could interfere with the regulation of these two molecules, namely focusing on physical exercise.

The SIRT family (SIRT1-7) consists of evolutionarily conserved NAD+-dependent deacetylases that play a role in various cellular metabolic processes by deacetylating target proteins [1]. Among the various SIRTs, SIRT 1 and 3 (SIRT1; SIRT3) are the most studied and are associated with mitochondrial biogenesis and mitophagy, which are considered protective factors in the aging process, such as in the cardiovascular system and in neurodegenerative diseases [1,5,6,7].

Regarding SIRT3, it is located within the mitochondria and its functions have not yet been completely understood. Findings suggest that SIRT3 deacetylates and activates the mitochondrial enzymes involved in fatty acid β-oxidation, amino acid metabolism, electron transport chain, and antioxidant defenses [8]. Some studies have demonstrated the positive effects of physical exercise in SIRT3. It seems that physical exercise can modulate the expression of SIRT3 in different tissues, such as increasing the SIRT3 activity in neurons, bone, and muscle [3,5,9]. Considering the important and positive effects of regular physical exercise on mitochondria, such as counteracting age-related effects [10,11,12], it seems to make sense that the improvement in mitochondrial function should be mediated by the upregulation of SIRT3.

The mammalian target of rapamycin (mTOR) integrates several signaling processes such as protein synthesis and anabolic metabolism, and coordinates cell growth, proliferation, and fate decisions. It has also been extensively described to be altered in various types of cancer as a regulator of proliferation, growth, metabolism, angiogenesis, and cell metastasis [13]. There are two mTOR complexes: mTOR complex 1 (mTOR1), which promotes increased protein, lipid, nucleotide, and ATP production, and general catabolic inhibition; and mTOR complex 2 (mTOR2), which appears to promote the reorganization of the actin cytoskeleton and plays an important role in chemotaxis and migration [13]. This signaling pathway is also shown to be an important regulator of glucose metabolism and lipogenesis, as the articulation between the complexes provides a protective mechanism, such as in insulin regulation [14]. In addition to the several relevant functions of mTOR in the body, it has also been also associated with the aging process. Indeed, in some model organisms, such as in C. Elegans, Drosophila melanogaster, and in mice, interventions have shown that the suppression of mTOR could expand the maximal life span [15,16,17,18]. Also, the dysregulation of mTOR pathways has been observed in different types of cancers [19,20]. Additionally, some of the drugs used in therapies for cancer inhibit the proliferation of the tumor cells by the mTOR pathway. Furthermore, mTOR, defined as a protein signaling pathway, has the ability to promote muscle hypertrophy at the skeletal muscle level [21,22]. The literature is not entirely consistent regarding the optimal type, intensity, and volume of exercise necessary to achieve the best adaptation. Mazo et al. [23] conducted a study on untrained men to analyze the effects of aerobic and resistance training on mTOR activation. The study concluded that both modes of exercise resulted in similar increases in mTOR signaling during the early post-exercise timeframe.

A study by Shirai et al. [24], in rats, aimed to evaluate the influence of the concurrent training order in mTOR activation. The authors concluded that resistance training before aerobic training seems to be more efficient in activating mTOR and mitochondrial biogenesis, although aerobic training before resistance training also proved effective in biogenesis.

Jones et al. [25] conducted a study in 30 men trained in the sport of cycling in order to understand the acute physiological effects of concurrent training. The results are in accordance with the aforementioned studies, refuting the premise that concurrent training could have antagonistic and inhibitory effects on protein signaling pathways and mitochondrial biogenesis. Therefore, the literature begins to show that this type of training does not interfere negatively with hypertrophy or adaptations at the mitochondrial level.

Oxidative stress, inflammation, and mitochondrial dysfunction should also be considered with regard to the activation of mTOR. The literature has shown that exercise promotes beneficial organic adaptations, including an anti-inflammatory state and a positive balance between reactive oxygen species production and antioxidant capacity [10,26,27]. Indeed, a 16-week intervention program of combined aerobic and strength exercises, comprising a control and an exercised group, in a sample population over 40 years old, showed that physical exercise could promote several positive changes in oxidative stress balance, namely in DNA damage protection in lymphocytes [10]. It is also mentioned in the literature that mTOR signaling modulates T cell development, activation, differentiation, memory generation, and function through a diverse set of transcription factors highlighting the complexity of the involvement of mTOR in lymphocyte biology [2].

Considering the numerous organic functions that are regulated by SIRT and mTOR, as well as the effects of physical exercise on these intracellular molecules, particularly in muscle, this research aimed to study the effects of an 8-week combined physical exercise program on SIRT3 and mTOR levels in lymphocytes in men.

Given the previously mentioned interplay between oxidative stress, inflammation, mitochondrial dysfunction, and mTOR activation, along with the specific effects of different exercise types on these mechanisms, there is a need for a comprehensive understanding of how exercise, specifically combined high-intensity interval training and aerobic exercise, influences the expression and functions of SIRT and mTOR in human lymphocytes. Therefore, this research aimed to study the effects of an 8-week combined physical exercise program on SIRT3 and mTOR levels in lymphocytes in men, and also in lipid peroxidation.

## 2. Materials and Methods

### 2.1. Participants

A group of nine Caucasian men, with ages ranging from 56 to 73 (mean = 61.2; SD = 5.4), underwent an 8-week training protocol involving combined physical exercise. Prior to their participation in the study, all candidates underwent thorough screening by a physician. Additionally, they participated in an individual face-to-face interview (questionnaire) to discuss their lifestyle habits. Inclusion criteria for the study were men over 45 years old without any health conditions that could interfere with the protocol tests or the physical exercise program. Exclusion criteria included individuals with metallic prosthesis implants, artificial pacemakers, those who required walking assistance, and those with known metabolic or endocrine disorders affecting the musculoskeletal system. The experimental procedures adhered to the principles of the Helsinki Declaration (UNESCO Universal Declaration on Bioethics and Human Rights, 2006) and received approval from the Ethics Committee of the Research Centre in Sports, Health, and Human Development at the University of Trás-os-Montes and Alto Douro (reference number 052012). Written informed consent was obtained from each participant, granting permission to use their information for the present report.

### 2.2. Testing Procedures

Before and after the experimental period (pre- and post-training), the tests were administered under the supervision of the same researchers. Subjects were directed to observe an 8 to 9 h fasting period before blood collection. Furthermore, they received instructions to adhere to their usual routines, encompassing sleep, diet, and daily physical activity, particularly in the 24 h leading up to the collections.

#### 2.2.1. Blood Sample Collection

Between 8:30 a.m. and 10:00 a.m., in fasting conditions, a venous blood sample (10 mL) was taken from each subject. The samples were collected in EDTA test tubes to prevent coagulation and processed within the following 19 min.

#### 2.2.2. Plasma Separation and Lymphocytes Isolations

A blood sample of approximately 10 mL was combined with cold phosphate-buffered saline solution (PBS) in a 1:1 ratio. After gentle mixing, the resulting mixture was layered onto 20 mL of Lymphoprep in a 50 mL conical plastic tube and centrifuged for 20 min at 2000× *g* rpm. Subsequently, around 4 mL of the plasma band was carefully collected into a 2 mL Eppendorf tube and promptly frozen at −80 °C for future analysis.

Simultaneously, the lymphocyte layer was extracted using a Pasteur pipet and transferred to a plastic centrifuge tube containing 10 mL of cold PBS. Following mixing, the sample underwent centrifugation for 10 min at 2000× *g* rpm. The resulting pellet, comprising lymphocytes from the band, was resuspended in 10 mL of cold PBS and frozen at −80 °C for subsequent analysis.

#### 2.2.3. Total Protein Determination

Spectrophotometric estimation of total protein in plasma was conducted using the biuret method, with serum albumin serving as the standard [28].

#### 2.2.4. Lipid Peroxidation

The malondialdehyde (MDA) assay was employed to measure the non-specific lipid peroxidation level in plasma. This involved determining the levels of lipid peroxides as the quantity of thiobarbituric acid reactive substances (TBARS) formed, following the method outlined by Wills [29] with some modifications. In this procedure, 100 μL of plasma samples were mixed with 200 μL of trichloroacetic acid (10%) and centrifuged at approximately 15,000× *g* for 1 min. Subsequently, 200 μL of the supernatant were combined with 200 μL of thiobarbituric acid (TBA) reagent (1% thiobarbituric acid). The mixture underwent heating at 80–90 °C for 10 min and was then cooled to room temperature for 20 min. The estimation of lipid peroxidation was based on the appearance of MDA, which was quantified spectrophotometrically by measuring the absorbance at 535 nm. The amount of MDA formed was calculated using a molar extinction coefficient (ε) of 1.56 × 105 M^−1^ cm^−1^, and the results were expressed as MDA concentration (nmol mg^−1^ of protein).

#### 2.2.5. SIRT3 and mTOR Activity Assay

Western blot was performed, which allows for the identification and relative quantification of proteins in biological samples, whose antibody–antigen specificity allows target proteins to be identified in a complex protein mixture, after their separation by molecular weights [30]. For this purpose, the indirect detection method was used.

Previously isolated lymphocytes were thawed, resuspended, and subsequently centrifuged (100 μL of sample for 5 min at 500× *g*, at 4 °C) in a refrigerated centrifuge, and the supernatant was discarded. Samples were lysed with SDS-PAGE loading buffer (0.125 M Tris-HCl at pH 6.4; 10% glycerol; 4% SDS (*w*/*v*); 10% 2-mercaptoethanol; and 0.001% bromophenol blue). Samples were heated for 5 min at 95 °C in a thermoblock and, after cooling, centrifuged again (9500× *g*, 5 min).

For electrophoresis, two different gels were prepared: the running gel—10% SDS polyacrylamide prepared with 3.95 mL of distilled water, 3.35 mL of 30% bis-acrylamide, 2.5 mL of 1.5 M Tris-HCl (pH 8.8), 0.1 mL of 10% SDS (*m/v*), 0.1 mL of 10% AMPS (*m/v*), and 0.004 mL of TEMED—and the stacking gel—prepared with 2.8 mL of distilled water, 0.85 mL of 30% bis-acrylamide, 1.25 mL of 1.5 M Tris-HCl (pH 6.8), 0.05 mL of 10% (*m/v*) SDS, 0.05 mL of 10% (*m/v*) AMPS, and 0.01 mL of TEMED.

In each well, 30 μL of the samples were placed, and 12 μL of a molecular weight marker (Prestained Protein Marker, Broad Range (11–190 kDa), Cell Signalling Techonology; Izaza Scientific, Porto, Portugal) was added to the first column, allowed to run at 60 V for 10 min using a Tetra Cell Mini-PROTEAN (Bio-Rad Laboratories, Lda., Algés, Portugal), then switched to 100 V for another 90 min.

After finishing the electrophoresis, the proteins were transferred from the gel to a polyvinylidene fluoride membrane (PVDF) previously activated with methanol and equilibrated in transfer buffer. The transfer was performed at 100 V for 120 min in ice-cooled transfer buffer.

After transfer, the non-specific binding sites were blocked with 5% (*w*/*v*) skim milk powder in TBS-T buffer (20 mM Tris-HCl, 137 mM NaCl, and 0.1% (*v*/*v*) Tween-20, pH 7.6) for 60 min. After blocking, the membranes were washed with TBS-T three times (15 min + 5 + 5) and incubated overnight at 4 °C with the following primary antibodies: β-actin (1:750, mouse anti-human, sc-47778, Santa Cruz Biotechnology; Frilabo, Oeiras, Portugal), sirtuin 3 (1:1000, rabbit anti-human mAb, C73E3, Cell Signalling Techonology), and mTOR (1:1000, rabbit anti-human mAb, 7C10, Cell Signalling Technology).

After incubation, the membranes were washed again with TBS-T three times (same protocol) to remove excess antibody and reduce non-specific labeling, and incubated with the respective secondary antibody (1:1000, goat anti-rabbit or goat anti-mouse alkaline phosphatase linked antibody, GE-Healthcare) for 1 h at room temperature.

There was another wash with TBS-T, three times (same protocol), and the bands were revealed with a chemofluorescent substrate (ECF) and visualized using a Gel DocTM EZ Gel Documentation System (Bio-Rad Laboratories, Lda., Algés, Portugal). Relative quantification of sirtuin 3 and mTOR was performed with Image Lab 5.1 software (Bio-Rad Laboratories, Lda., Algés,, Portugal), and normalized using β-actin as the “housekeeping” protein.

### 2.3. Physical Exercise Training

Over the course of 8 weeks, the subjects engaged in exercise training, with three sessions per week, each lasting 45–60 min and conducted on non-consecutive days. Each session comprised three components: 20 min of aerobic exercise, 20–30 min of high-intensity interval training (HIIT), and 5–10 min of stretching and cool down. The aerobic exercises included walking, running, biking, rowing, and elliptical workouts. The intensity of the aerobic component was progressively adjusted within the range of 55% to 75% of the heart rate reserve (HRR) calculated using the formula HRR = 207 − (0.7 × Age) [31]. Subjects were monitored using a Polar S625X (Kempele, Finland) cardiac monitor. The HIIT workout included mainly body weight exercises, but also using elastic bands, steps, bars, and dumbbells. The HIIT training intensity was progressively increased along the program. The workout periods increased by 20 s at the beginning of the program to 40 s at the end of the program. Subjects were encouraged to achieve submaximal efforts during the HIIT training.

### 2.4. Physical Testing

Before performing the physical tests, a specific warm-up was allowed, consisting of a 5 min full body mobility workout using body weight. Subsequently, subjects executed the physical tests, with procedures explained before each test.

For the vertical jump assessment, a trigonometric carpet (Ergojump Digitime 1000; Digitest, Jyvaskyla, Finland) was utilized to measure maximum height in the counter-movement jump (CMJ). Each subject initiated the jump from an erect standing position, and the end of the concentric phase coincided with full leg extension (180°). The test was conducted three times, with a 2 min rest period between each attempt, and the best result of the three attempts was considered.

The 20 m velocity test involved walking (not running) in a single maximum sprint over 20 m, with the time recorded. Subjects started from a stationary position, with one foot behind the starting line and in front of the other. Participants were encouraged to maximize speed, driving hard with their arms and legs, and to continue walking hard until reaching the finish line.

For the upper body muscular strength assessment, the medicine ball throwing performance was evaluated using a 2 kg medicine ball (MBT) (Ø 0.60 m). Each subject, seated on the floor with the posterior trunk region against the wall, held the ball to the front with both hands. Three approved attempts were made with 1 min rest intervals. The maximal throwing distance was measured using a flexible steel tape, and only the best attempt was used for further analysis [32].

### 2.5. Aerobic Capacity

The 6MWT (6-min walk test) is a practical and straightforward assessment that measures the distance a patient can quickly walk on a flat, hard surface within a 6 min timeframe. This test evaluates the global and integrated responses of various systems engaged during exercise, including the pulmonary and cardiovascular systems, systemic and peripheral circulation, neuromuscular system, and muscle metabolism [33].

It is important to note that the 6MWT does not offer specific insights into the functioning of individual organs and systems involved in exercise or the mechanisms of exercise limitation. The self-paced nature of the 6MWT assesses submaximal levels of functional capacity, as most patients do not reach their maximal exercise capacity during the test. Participants have the autonomy to choose their exercise intensity and can stop and rest as needed.

While the 6MWT may not measure maximal exercise capacity, it is valuable for assessing the submaximal level of functional capacity, which is more representative of daily physical activities. Since most daily activities are performed at submaximal levels of exertion, the 6MWT provides a meaningful reflection of functional exercise levels for everyday tasks.

### 2.6. Statistical Analysis

To assess data distribution, the Shapiro–Wilk test and box plot analysis were employed. For variables with a normal distribution, a paired sample t-test was utilized to compare the results between pre- and post-training. In the case of non-normally distributed variables, particularly the velocity measure, the Wilcoxon signed rank test was applied. The significance level was set at 0.05. Data analysis was conducted using the software ‘Statistical Program for Social Sciences—SPSS’ version 27.0.

## 3. Results

Physical performance and anthropometric characteristics are presented in Table 1. Interestingly, although not statistically different, a post-test revealed an increasing trend for weight (kg: *p* = 0.273, d = 0.61373) but a decreasing trend for waist circumference (cm: *p* = 0.075, d = 2.19522), which could indicate a slight positive effect on body composition from the 8 weeks of training in terms of decreased waist circumference (102.4 cm to 100.4 cm). Regarding physical performance, after the physical exercise program, positive and significant results were found, such as an increase in aerobic capacity, evaluated by 6MWT (*p* = 0.002, d = 40.95492), and in velocity test (20 m sprint, *p* = 0.055, d = 0.29933). Regarding CMJ (*p* = 0.096, d = 0.02080) and medicine ball throwing (*p* = 0.662, d = 0.40820), no differences were found after the physical program.

Concerning oxidative stress, inflammation, and mitochondrial dysfunction, Table 2 describes the main values of MDA concentration (µmol MDA/mg protein), and relative amounts of SIRT3 (SIRT3/β-actin) and mTOR (mTOR/β-actin).

**Figure 1:**
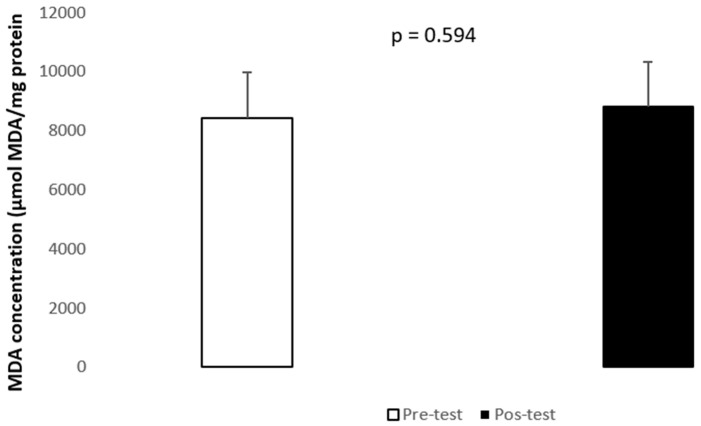
Quantification of MDA, as biomarker of oxidative stress. Mean (±SD) MDA concentration in the participants before (1) (□) and after (2) (■) the 8-week training period.

**Figure 2:**
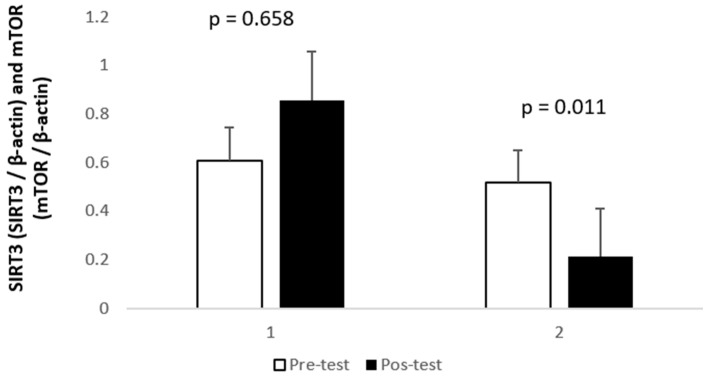
Relative quantification of SIRT3 (1) and mTOR (2) levels in lymphocytes, as denoted. See methods for details; mean (±SD) measured in lymphocytes of blood samples collected in the participants before (□) and after (■) the 8-week training period.

Regarding lipid peroxidation (Figure 1), evaluated through MDA concentration, no differences were found after the exercise program (*p* = 0.594, d = 4188.211), with levels from 8419.9 to 8799.9 nmol MDA/mg protein, between pre- and post-test. A statistically significant decrease was found in the relative abundance of mTOR (z = −2.533; *p* = 0.011, d = 0.58540) (Figure 2). Also, there was a trend towards a decrease in the relative abundance of SIRT3 (*p* = 0.658, d = 0.602502), but not statistically different.

## 4. Discussion

The present study aimed to analyze the effect of 8 weeks of a combined physical exercise program on SIRT3 and mTOR concentration in human lymphocytes and plasma lipid peroxidation. Our main finding revealed that physical exercise induced a significant decrease in mTOR in lymphocytes. Moreover, no significant changes in SIRT3 in lymphocytes and plasma lipid peroxidation were found.

Considering the American College of Sports Medicine (ACCMs) recommendations, the combination of aerobic training and resistance training is an effective strategy to improve overall functional capacity and to promote health [34]. Regarding this, in our study, volunteers enrolled in this exercise training program showed significant improvement in their cardiorespiratory and velocity capacity. Performance in the squat jump showed a positive trend but no significant changes were found after the exercise program. It should be mentioned that considering the age of the sample, it was expected that joint weakness in some subjects might have compromised the improvement in the squat jump test and in ball throwing, and it is essential to take into consideration that this is a ballistic movement, which requires motor coordination dependent on specific neuromuscular activation and technical skills [35], abilities that were not the main focus of the implemented exercise program.

Regarding mTOR, its functions on the dynamics of the immune cell system, such as proliferation, growth, and activation have recently been investigated. Indeed, mTOR signaling has been linked to the T cell biology dynamics, ranging from T cell development and activation to lineage specification and memory maturation [2]. In addition, it seems that many of these physiological effects are mediated by transcription factors, and its expression or activity is dependent upon mTOR activation [36,37]. Our results showed that 8 weeks of physical exercise contributed to decreased mTOR in lymphocytes. Regarding our results, despite the aforementioned increase in mTOR in muscle as a consequence of physical exercise observed in some studies, it is not clearly expected to observe the same effect in other tissues or from a systemic perspective. Aerobic exercise, and to a lesser degree, resistance exercise—both of which impose substantial metabolic stress—could lead to the activation of AMPK (AMP-activated protein kinase), subsequently inhibiting mTOR. This effect may occur not only in muscular tissues but also in non-muscular tissues such as the liver, fat, and tumor tissues.

Indeed, while mTOR signaling plays a crucial role in controlling the immune system, the hyperactivation of mTOR1 not only hinders cells transitioning into a memory state but has also been linked to general inflammation, cardiovascular disease, and atherosclerosis [37]. Moreover, several works have demonstrated the positive anti-inflammatory effect of regular physical exercise [38,39]. Also, Agostini et al. (2018) [40] have reviewed and described the inhibitory effects of physical exercise on triple-negative breast cancer tissue. In this context, the known hormetic effect of exercise seems to regulate the immune dynamic homeostasis. Considering this, our results emphasize the importance of future studies exploring the role of regular physical exercise in regulating mechanisms underlying systemic inflammation, possibly mediated by mTOR and also by other related factors such as oxidative stress, antioxidant capacity, and mitochondrial function.

As mentioned before, of all the SIRT family, SIRT3 specifically has been described to be involved in several aspects of mitochondrial metabolism, functionality, and homeostasis, protecting mitochondria from different damages [1], and it was expected to increase after the exercise program. Nevertheless, our results did not reveal any statistically significant changes in SIRT3 as a consequence of physical exercise. Poor information has been published regarding the effects of exercise on SIRT3, particularly in human lymphocytes. Considering that SIRT3 plays an important role in regulating mitochondrial metabolism, these results suggests that the exercise program possibly did not induce an increase in mitochondrial content and oxidative capacity in lymphocytes, or if it occurred, the molecular mechanisms underlying changes were not dependent on the SIRT3 pathway. Indeed, our results evidenced an increase in aerobic capacity (6MWT), expressing an increase in overall oxidative capacity. According to this, several other works demonstrated that physical activity increases oxidative capacity not only in muscle but also in other tissues such as in lymphocytes [11,12,41,42]

Regarding the concentration of MDA, the results obtained in this study showed that the combined exercise program did not induce any significant changes. These findings are not consistent with previous studies [10,27]. Some possible explanations for these inconsistent results should be mentioned and discussed to better understand this theme. First of all, the duration of the previous studies was 16 weeks instead of 8 weeks in the present investigation, which could indicate that exercise protocols should be longer to achieve a better physiologic adaptation. Second, based on the results of our pre-test, it should be noted that our sample had a good level of aerobic capacity [43] and good lower limb strength [44]. Therefore, although the participants were not previously enrolled in a structured physical exercise program, they were not sedentary. Additionally, the antioxidant status, which is typically influenced by physical exercise and nutrition, was not measured. This could have provided insights into oxidative stress damage and the lack of change in SIRT3. This study has some limitations, specifically the number of participants, that only a few finished and completed all the exercise programs and evaluations, and in the future, participants should be analyzed for a long period during the exercise training and detraining.

## 5. Conclusions

In conclusion, the 8-week combined physical exercise program had notable effects in some of the parameters studied, particularly on the mTOR concentration in lymphocytes. The significant decrease in mTOR suggests a potential role of exercise in modulating immune cell dynamics. Considering the positive effects of regular physical exercise as an anti-inflammatory, described in the literature, our results emphasize the need for future studies to delve deeper into the mechanisms underlying systemic inflammation, possibly mediated by mTOR. While the exercise program positively impacted cardiorespiratory and velocity capacity, the lack of significant changes in SIRT3 in lymphocytes raises questions about its role in mitochondrial metabolism and oxidative capacity in this context. Despite the observed increase in overall oxidative capacity, the molecular mechanisms underlying these changes may not be dependent on the SIRT3 pathway. The study results regarding plasma lipid peroxidation, measured through MDA concentration, did not show significant changes after the 8-week exercise program. This contrasts with some previous studies, and potential explanations include the shorter duration of the exercise protocol compared to prior research and the good baseline aerobic capacity and lower limb strength of the participants. In summary, this study contributes valuable insight into the complex interplay between exercise, molecular pathways (mTOR and SIRT3), and oxidative stress markers. It highlights the need for further research, considering factors such as exercise duration, baseline fitness levels, antioxidant status, and inflammation variables to better understand the nuanced effects of physical activity on cellular and systemic health.

## Figures and Tables

**Table 1:** Mean values ($\bar{x}$ ± SD) of body weight and physical performance, before and after the physical program.

Variable	Pre-Test $(\bar{x}$ ± SD)	Post-Test $(\bar{x}$ ± SD)	*p* Value
Weight (kg)	79.7 ± 2.58	83.48 ± 5.93	*p* = 0.273
Waist Circumference (cm)	102.4 ± 3.97	100.4 ± 2.36	*p* = 0.075
Aerobic Capacity (6MWT) (m)	615.4 ± 45.3	694.2 ± 37.4	***p* = 0.002**
Medicine Ball	6.71 ± 0.67	6.62 ± 0.35	*p* = 0.662
Squat Jump (CMJ) (m)	0.17 ± 0.04	0.20 ± 0.05	*p* = 0.068
Velocity (sec)	4.7 ± 0.5	4.4 ± 0.3	***p* = 0.043** *

*p* ˂ 0.05, statistically significant differences between pre- and post-exercise training moments. * Data based on Wilcoxon signed rank test.

**Table 2:** Mean values ($\bar{x}$ ± SD) of MDA concentration (nmol MDA/mg protein), SIRT3 (SIRT3/β-actin), and mTOR (mTOR/β-actin), before and after the physical program.

Variable	Pre-Test $(\bar{x}$ ± SD)	Post-Test $(\bar{x}$ ± SD)	*p* Value
MDA (nmol MDA/mg proteín)	8419.9 ± 4615.8	8799.9 ± 3163.43	*p* = 0.594
SIRT3 (SIRT3/β-actin)	0.609 ± 0.404	0.516 ± 0.390	*p* = 0.658
mTOR (mTOR/β-actin)	0.857 ± 0.593	0.214 ± 0.097	***p* = 0.011**

*p* ˂ 0.05, statistically significant differences pre- and post-exercise training moments.

## Data Availability

The data presented in this study are available on request from the corresponding author.
